# Lateral Compression Fragility Fractures of the Pelvis: Diagnosis, Classifications, and Modern Management

**DOI:** 10.1007/s11914-024-00891-1

**Published:** 2024-09-23

**Authors:** Joseph T. Patterson, Joshua A. Parry

**Affiliations:** 1 Department of Orthopaedic Surgery, Keck School of Medicine of University of Southern California, 1520 San Pablo Street, Suite 2000, Los Angeles, CA 90033-5322 USA; 2 Denver Health Medical Center, University of Colorado School of Medicine, Denver, CO USA

**Keywords:** Pelvis, Fracture, Fragility, Lateral compression, LC1, Older adult, Review

## Abstract

**Purpose of Review:**

To describe the diagnosis, classification, and modern management of lateral compression fragility fractures of the pelvis.

**Recent Findings:**

Practice patterns are shifting toward early operative treatment of fragility fractures of the pelvis among patients who are unable to mobilize or whose injuries demonstrate occult instability on stress imaging. Early internal fixation appears to decrease pain, facilitate mobilization, accelerate hospital discharge, and minimize morbidity in this population.

**Summary:**

Lateral compression pelvic ring injuries are the most common type of fragility fracture of the pelvis. Similar to fragility fractures of the hip, lateral compression fragility fractures of the pelvis are typically sustained in a ground level fall. These injuries are associated with long acute hospital and post-acute facility admissions, loss of physical function, loss of independence, mortality, anxiety, sleep disturbance, and caregiver burnout. Unlike hip fractures, for which urgent operative treatment and early mobilization reduce mortality, lateral compression fragility fractures of the pelvis are commonly treated without surgery. Recommendations for nonoperative management of these injuries in older adults may be inappropriately generalized from studies of younger patient populations with high-energy mechanisms of pelvis fracture. However, strong evidence to support early internal fixation of these injuries practice is lacking. High quality investigations of early surgical intervention for lateral compression fragility fractures of the pelvis are needed to guide care for these patients.

## Introduction

Fragility fractures of the pelvis affect approximately 600,000 adults each year [1–3]. The incidence and costs of fragility fractures of the pelvis are rising faster than other fragility fractures of the hip, spine, and wrist [2–4]. Lateral compression (LC) type pelvic ring injuries are the most common type of fragility fracture of the pelvis, making up 80% of pelvic fractures in this population [5]. Older adults typically sustain these injuries in a fall, with the ground imparting a lateral force on the pelvis and failure occurring through metabolically-impaired bone of one or more segments of the pelvic ring.

Like fragility fractures of the hip, nearly all older adults who sustain an LC fragility fracture of the pelvis experience serious risks of death, sustained functional decline and disability, and loss of independence. Over 90% of patients require a hospitalization averaging greater than one month, 25–36% of patients do not discharge directly home, 100% of patients require an assistive device at discharge to ambulate and 50% of patients require assistance from another person for mobility [6–8]. Mortality at one year is approximately 14 to 27% [6–8]. Survivors report anxiety, sleep disturbance, sexual dysfunction, financial strain, and caregiver burnout as direct consequences of their pelvic fracture [9].

Unlike hip fractures however, for which urgent surgical intervention and mobilization clearly reduce mortality and disability, the management of LC pelvic ring fragility fractures is often nonoperative [10, 11]. Acceptable outcomes have been observed with nonoperative care of LC fracture patterns sustained with high-energy injury mechanisms fracture [11–13]. However, nonoperative management of LC pelvis fragility fractures in older adults may represent an inappropriate generalization of observations made in younger patient samples with different mechanisms of injury.

The nonoperative treatment of LC pelvis fragility fractures in older adults has been challenged, with some authors arguing for more aggressive management of these patients, similar to hip fractures [14–17]. Stress imaging, such as examination under anesthesia (EUA) or stress radiographs in the emergency department, has been proposed as one potential way to identify patients experiencing pain due to fracture instability who would potentially benefit from early surgical fixation [18–21]. However, there is little agreement on the use of stress imaging, the need for surgical fixation, or type of fixation constructs to use when operative treatment is utilized [22, 23].

The purpose of this article is to review the current state of the art of evaluation and management of LC fragility fractures of the pelvis, including diagnosis and classification, the use of stress imaging to identify occult instability, decision making for operative versus nonoperative management, and the types of fixation constructs used for surgical stabilization.

## Definition and Classifications

LC fragility fractures of the pelvis are caused by low-energy mechanisms that impart a laterally directed force at the level of the hip in patients with poor bone quality [24]. The mechanism of injury is most commonly a ground-level fall from standing, although fragility fractures of the pelvis may also be also observed following falls while bicycling and skiing, as well as low speed collisions between pedestrians and motorized vehicle [1, 25, 26].

Lateral compression type pelvis fractures were originally described by Pennal and colleagues, who distinguished these patterns from patterns observed with anterior–posterior compression and vertical shearing mechanisms [27]. Tile and co-authors refined this concept with a distinction between complete and incomplete injuries of the posterior based on stability to rotational and vertical stresses [28]. Young and Burgess further described a spectrum lateral compressions of increasing instability to internal rotation stress as type 1 (LC1), type 2 (LC2), and type 3 (LC3) injuries [24, 29]. The LC1 injury pattern includes rami fractures anteriorly and a unilateral sacral fracture posteriorly, which may be complete or incomplete. The LC2 injury pattern involves a posterior fracture through the iliac wing (crescent fracture) rather than through the sacrum. The LC3 injury pattern is either an LC1 or LC2 injury in addition to an external rotation injury of the contralateral posterior ring, resulting in disruption of the contralateral anterior sacroiliac joint ligaments with increasing degrees of instability. These descriptions were incorporated into the AO/OTA fracture classification as subgroups [30].

A limitation of the Young Burgess classification, however, is the wide spectrum of injuries included under the LC1 type, ranging from fractures with nondisplaced incomplete sacral fractures to severely displaced complete sacral fractures, making the classification insufficient to guide management [1]. Sagi et al. subclassified the LC1 pattern to account for injury severity among LC injuries with minimally displaced posterior ring fractures by identifying occult instability with an examination under anesthesia (EUA). LC1 fractures that displaced ≤ 1 cm on fluoroscopy were defined as LC1a and managed nonoperatively while injuries that displaced > 1cm were defined as LC1b and treated with operative fixation [31]. Tucker et al. further modified this classification scheme by defining three distinct LC1 types of increasing severity and associated treatment recommendations: LC1a (< 1 cm of fracture displacement on injury radiographs and < 1 cm of dynamic displacement on stress imaging; nonoperative), LC1b (< 1 cm of fracture displacement on injury radiographs and ≥ 1 cm of dynamic displacement on stress imaging; operative), and LC1c type (≥ 1 cm of fracture displacement on injury radiographs; operative) [32]. The AO/OTA fracture classification distinguishes between LC1 fractures with and without rotation instability, but offers no definition or guidance regarding how this instability should be determined [30].

Rommens et al. developed the Fragility Fracture of the Pelvis (FFP) classification to incorporate pelvic fragility fracture types that do not clearly fit the Young Burgess or AO/OTA classification systems and to also guide management [33]. This fracture classification has 5 types of injuries with 11 total subtypes. Type I injuries include isolated rami fractures without posterior injury, for which nonoperative management was recommended. Type II injuries include fractures with anterior fractures and nondisplaced posterior injuries, for which a trial of nonoperative management is recommended, followed by fixation if the patient was unable to mobilize after a week. Type III to IV injuries include displaced posterior injuries for which anterior and posterior pelvic ring fixation is recommended. The FFP classification, similar to the original Young Burgess classification, does not account for occult instability on stress imaging nor does the FFP classification guide treatment decisions based on instability examinations.

## A Role for Advanced Imaging?

Plain radiographs are the standard diagnostic screen test for identifying fragility fractures of the pelvis. Advanced imaging is often obtained, most commonly computed tomography (CT) of the pelvis without contrast. CT of the pelvis may assist with classification of the fracture pattern [33] and with identifying features that may predict fracture instability [34]. Almost all (97%) patients presenting with pubic rami fractures have a posterior ring injury on CT scan [35]. A lack of posterior ring injury on CT scan also does not rule out the presence of a fracture as the sensitivity for sacral fractures has been shown to be 66% to 75% [36, 37]. Nüchtern et al. evaluated isolated anterior pelvic fractures with CT and MRI scans and reported that 17% of patients had a posterior ring injury that were not present on CT scan [38].

However, the clinical utility of advanced imaging and the clinical significance of an occult posterior ring fracture are controversial. CT of the pelvis does little to improve agreement between pelvic trauma surgeons on fracture type or treatment [39]. Natoli et al. observed that among 87 patients ≥ 60 years old who received a CT or magnetic resonance imaging (MRI) of the pelvis for diagnosis of a low-energy traumatic pelvic ring injuries, the presence of a posterior pelvic ring injury on the advanced imaging was not associated with displacement > 1 cm at presentation or at 6-week follow-up following nonoperative treatment with full weightbearing permitted [40]. Conversely, Tucker et al. [41] reported that 42% (8/19) of geriatric patients presenting with isolated rami fractures on CT scan and difficulty mobilizing secondary to pain had occult instability on stress radiographs. Six of the patients with occult instability received an MRI and an occult posterior ring fracture was identified in all six, suggesting that isolated rami fractures in older adults failing to mobilize may warrant stress imaging. Rommens et al. reported significant morbidity in patients admitted to the hospital with isolated pubic ramus fragility fractures, with these patients having a 4% in-hospital mortality rate, a 17% one-year mortality rate, and the proportion of surviving patients living at home dropped from 81 to 65% [42]. It is likely that many of these patients, based on the findings of the aforementioned studies, were LC1 injuries with occult instability, potentially explaining the out-of-proportion morbidity of seemingly benign isolated anterior rami fractures.

If surgery is indicated, advanced imaging is helpful for surgical planning. A detailed surgical plan includes implant trajectories, diameters, and lengths for percutaneous internal fixation based on CT- or MRI-based measurements of osseous screw corridors [43]. Identifying sacral dysmorphism or unusually pelvic tilt may inform alternate positioning to achieve satisfactory intraoperative imaging such as retroverting the pelvis to clearly observe and avoid implant violation of the sacral neuroforaminal tunnels [44], as well as selecting fixation strategies to avoid neurologic injury during guidewire, drill, and implant implacement [45].

## Stress Imaging

**Fig. 1:**
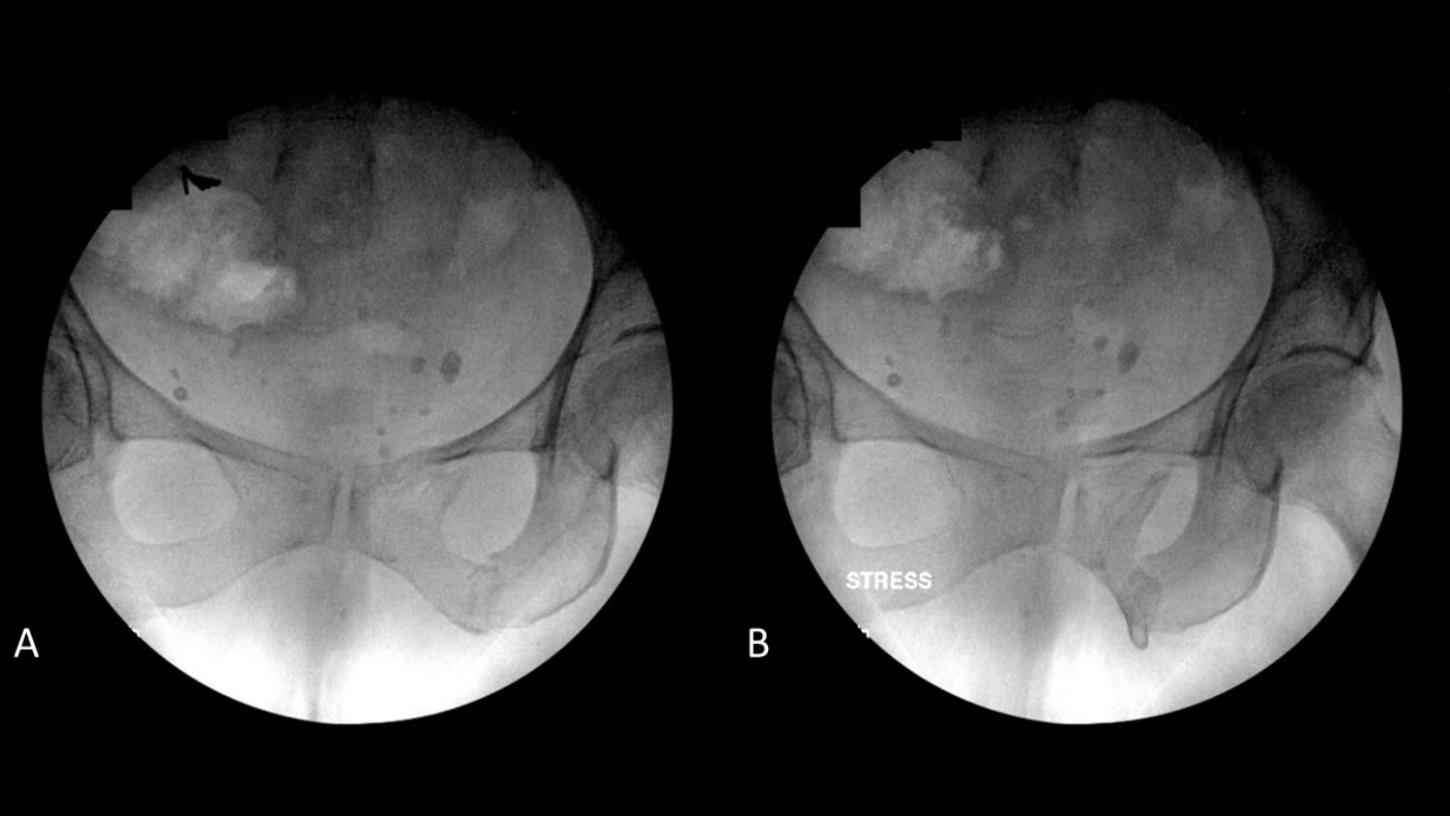
**(A)** Non-stress and **(B)** stress fluoroscopic images from an examination under anesthesia of a 77-year-old woman with a minimally-displaced LC1 fragility fracture of the pelvis demonstrating gross occult instability

The use and usefulness of stress imaging for guiding treatment decisions for minimally displaced LC injuries with minimally displaced posterior ring injuries are also controversial [1]. Multiple studies have demonstrated that these injuries have low rates of fracture displacement and high rates of union, however when these do occur, they are difficult to treat [11, 12, 46–48]. As a result, examination under anesthetic (EUA) was described as a method to identify fractures with occult pelvic ring instability that may be at higher risk for subsequent fracture displacement and/or poor outcomes. Kanakaris and colleagues described the use of an internal rotation stress examine under anesthesia in 40 patients with LC1 pelvis fractures. 23 patients (58%) demonstrated > 2 cm of displacement on fluoroscopic manipulation and were treated with sacroiliac screws in combination with retropubic screws, external fixator or anterior pelvic plating. Tsounidis et al. expanded this series, observing that patients who demonstrated > 2cm displaced and were managed surgically experienced less pain at 72 h, shorter hospital stay, shorter time to pain-free mobilization, and decreased opioid use [49]. Sagi and colleagues independently and concurrently described a more thorough EUA of the pelvis including compression over the trochanters to detect instability to internal rotation forces and a push–pull exam of the lower extremities to detect vertical instability on AP, inlet, and outlet images (Fig. 1) [31]. This group reported that 35% (7/20) of minimally displaced LC1 fractures displaced > 1 cm on fluoroscopy with EUA and were treated with operative fixation.

While EUA of the pelvic ring has been shown to improve the agreement among surgeons in regards to fracture stability and the need for surgical intervention, the technique has limitations [22]. Utilization of an operating room for an examination without treatment, as ostensibly occurs when the examination is negative, incurs a substantial resource burden for a hospital, requiring anesthetic materials and personnel, perioperative staff, transport, and at least one orthopaedic surgeon for the examination. The patient is exposed to an anesthetic, and thus risks including mortality, which may not be medically necessary. For these reasons, use of EUA is typically restricted to certain fracture patterns that are considered to have a high pre-test probability of instability (i.e. complete sacral fractures, high-energy mechanisms, bilateral rami fractures) [31, 46]. EUA also relies on compression applied by an examiner, which is likely variable between examiners and examinations, and requires interpretation of displacement on fluoroscopic imaging, which may be difficult to objectively measure while performing the EUA. EUA, as with all manual stress examinations, require radiation of healthcare personnel, which violates the “as low as reasonably achievable” guiding principle of radiation safety [50].

**Fig. 2:**
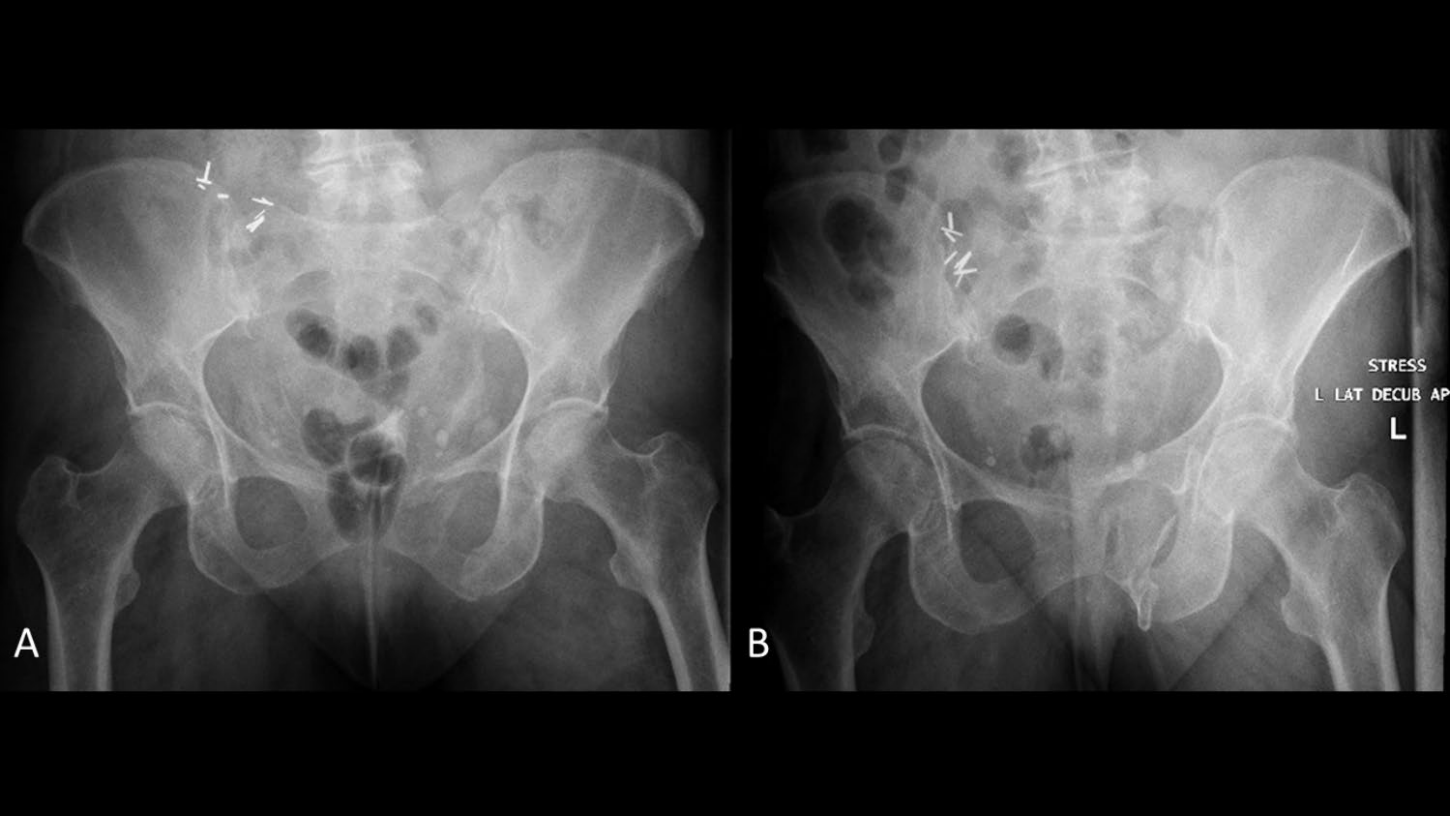
**(A)** AP pelvis radiograph and **(B)** lateral stress radiograph of the same patient from Fig. 1 redemonstrating the gross occult instability

The lateral stress radiograph, an anteroposterior (AP) pelvis radiograph taken in the lateral decubitus position, is an alternative to EUA that avoids many of the above limitations (Fig. 2) [18]. The lateral stress radiograph requires no anesthetic or sedation, can be done in the emergency department by radiologist technicians without physician supervision, and applies a relative standardized force using gravity and the patient’s body weight. Fractures can be objectively measured on the radiograph using the electronic imaging system and compared to standard supine AP pelvis radiographs to determine displacement. In a series of 131 consecutive patients receiving lateral stress radiographs, 62.5% (n = 80) of patients had occult instability, defined as ≥ 1 cm of dynamic displacement [32]. The median age of the cohort was 57 years, 60% were female, and 52% had low-energy mechanisms. Patients with occult instability, compared to those without, were older (63 vs 48 years; p = 0.009), more likely to have parasymphyseal rami fractures (74% vs 48%; p < 0.0001), and rami comminution (96% vs 38%; p < 0.0001). Occult instability had no association with high-energy injury mechanisms, sacral fracture completeness, or sacral fracture comminution. Limitations of the lateral stress radiograph include pain, with some patients being unable to tolerate the lateral position, the potential for malrotated radiographs that can make interpretation difficult, and the difficulty of obtaining in polytraumatized patients with long bone or spine fractures [51].

The emergency department (ED) supine stress radiograph utilizes the same exam as the EUA with an orthopaedic surgeon applying compression over the greater trochanters [19]. Radiography of the pelvis in the supine position avoids issues with malrotated radiographs and intolerance or inability of patients to be positioned lateral decubitus [51]. Like the EUA however, the ED stress radiograph relies on the force applied by the examiner. In a series of 70 consecutive patients evaluated with this technique, 19% had occult instability. The average age of the cohort was 59 years, 54% were female, and 48% had low-energy mechanisms. Fractures with occult instability, compared to stable injuries, were associated with higher energy mechanisms, complete sacral fractures, bilateral rami fractures, and parasymphyseal ramus fractures, and rami comminution, and had no association with age or gender. Interestingly, despite this series of patients having a similar distribution of age, gender, and low-energy mechanisms as the lateral stress radiograph series mentioned above, the rate of occult instability was much lower (17% vs 63%) and there were highly significant associations between occult instability and complete sacral fractures and high-energy injuries, not seen in the prior study. The stark difference in the rate of occult instability could be secondary to variability in the force applied by examiner, which could be biased by the clinical scenario. The association between occult instability and complete sacral fractures and high-energy mechanisms may also be biased by reviewers not being blinded to the presence of occult instability.

**Fig. 3:**
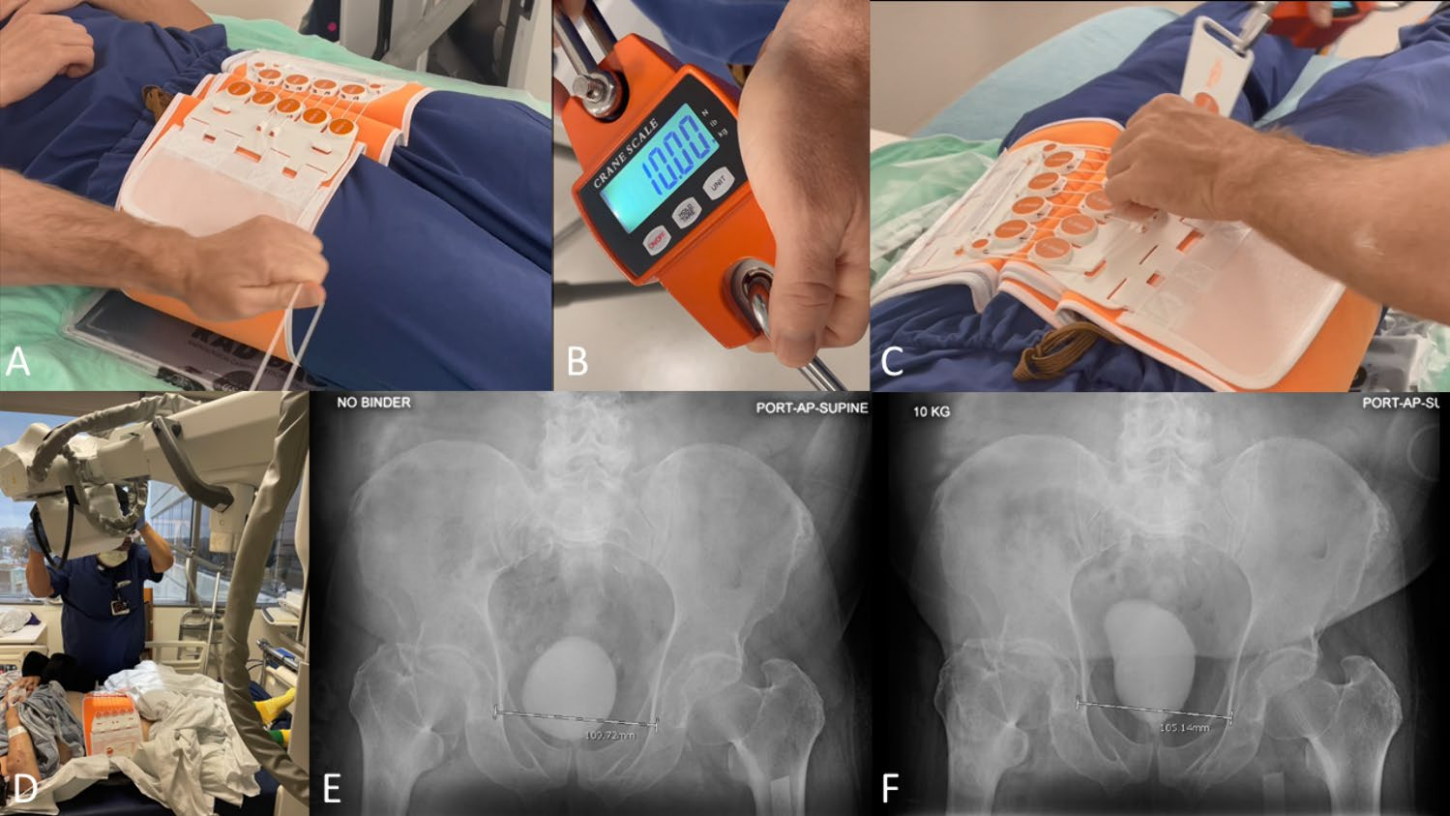
Photographs of surgeon **(A)** tensioning of a pelvic binder (B) using a hanging scale to a standardized force of 10 kg and **(C)** securing the binder in a compressed position. (D) Photograph of a 91-year-old-man with a minimally displaced LC1 fragility fracture of the pelvis comfortably undergoing pelvic binder radiography at 10 kg. **(C)** Non-stress and **(D)** stress radiographs of this patient’s fracture demonstrating 4 mm of displacement at 10 kg

Pelvic binder stress radiography (PBR) uses a circumferential pelvic compression device to apply a constant and reproducible force to the pelvic ring (Fig. 3). Similar to the lateral stress radiograph, PBR does not require an anesthetic. The binder may be applied point-of-care and tensioned to a specific force with a scale to minimize variability between examiners and examinations. Portable radiography in the supine position obviates the need patient transport or repositioning. In a cadaveric simulation, Patterson et al. demonstrated that 10 kg of applied force resulted in > 1 cm of displacement in 95% of simulated LC1 injuries and was able to discriminate between LC1a, b, c type variants [20]. A prospective clinical feasibility study of PBR enrolled 31 patients, all of whom tolerated the examination without sedation, anesthetic, or adverse event. Fracture displacement > 1cm was observed in 10 patients (32%) who subsequently received EUA and internal fixation, with PBR demonstrating 100% sensitivity and 100% specificity compared to EUA) [52].

The indications for stress radiography of LC1 pelvis fractures are evolving. Conventionally, a minimally displaced (< 1 cm or < 2 cm) pelvic ring injury with a complete sacral fracture is indicated for EUA by many pelvis surgeons [22, 23, 31, 49]. Determining completeness of sacral fractures on CT scans, especially in a geriatric population with poor bone quality, can be challenging. Hadeed et al. demonstrated that there was weak interobserver reliability for identifying complete sacral fractures among fellowship-trained orthopaedic trauma surgeons [53]. Four pelvic ring fractures with an incomplete sacral fracture (100% surgeon agreement) demonstrated occult instability on stress radiographs, indicating that completeness of a sacral fracture on CT scans does not rule out occult instability.

The interpretation of stress radiography of LC1 pelvis fractures remains highly controversial. At the time of publication there are no high quality definitive prospective data nor a consensus on a threshold of LC1 pelvis fracture stability beyond which a patient will experience an adverse outcome without stress examination or surgical intervention. Stress examinations inform the treatment recommendations for some surgeons and do not have a role in the management decisions made by others. The role of stress examination in treatment decisions for LC1 pelvis fractures warrants further study.

## Nonoperative versus Operative Management

Operative management is recommended for LC injuries with rotational or vertical instability secondary to disruption of the posterior ring [1]. However, much of the literature surrounding the operative versus nonoperative management of LC injuries involves younger patients with high-energy mechanisms and has limited applicability to LC fragility fractures. Sembler Soles et al. reviewed 118 patients with high-energy minimally displaced LC fractures [11]. The average patient age was 46 years and the average injury severity score (ISS) was 15; 99% of patients were treated successfully with nonoperative management with minimal fracture displacement. Only one patient converted to operative management due to fracture displacement and severe pain. This study was limited by the exclusion of patients who did not return to unassisted ambulation at last follow-up however, which could introduce selection bias.

Hagen et al. compared patients with high-energy LC1 and LC2 fractures treated with and without operative fixation at a major level-one trauma center, including polytraumatized patients with lower extremity injuries [54]. Most of the operative patients had fractures with > 1cm of displacement on static radiographs while nonoperative patients tended to have less severe, minimally displaced pelvis fractures. The average patient age was 35 in the operative group and 41 in the nonoperative group. They reported no clinically relevant differences in opioid use in the first 48 h or time to mobilization between operative and nonoperative groups.

Tornetta et al. performed a multi-center retrospective study including 194 high-energy LC patients [55]. The average patient age was 39 years and 74% were treated operatively. Patients treated nonoperatively had higher pain scores at 24 h and 3 months after the injury, but the differences were also small and unlikely to be clinically relevant.

Unlike the above studies, Rommens et al. evaluated operative versus nonoperative treatment of patients with pelvic fragility fractures classified using the FFP system [14]. Type II injuries (i.e. minimally displaced LC1s) were treated conservatively unless the patient was unable to mobilize secondary to pain after 3–5 days. They reported a median time to surgery of 6 days and a median hospital length of stay of 12 days with only half of patients being able to discharge home. When analyzing the 238 patients with type II fractures, 21.8% ultimately converted to surgery. These patients had a longer hospital length of stay (17 vs 9 days), were less likely to discharge home, but had a lower mortality rate.

Protocols based the ability of a patient to mobilize may obscure potential time-dependence in the treatment effect of surgery. As with hip fracture patients who experience worse outcomes and greater mortality with delays in surgical care [56], patients with LC1 pelvis fragility fractures who are functionally bedridden due to pain may not receive the same magnitude of benefits from internal fixation of the pelvis fracture a few days after injury compared with early fixation. Occult instability on stress imaging has been shown to be strongly associated with the inability to mobilize within the first 3 hospital days and may be used to identify and treat patients who will not be able to mobilize earlier [57]. Studies that do not obtain stress imaging are unable to differentiate between the outcomes of patients with occult instability and those without, which is a major limitation of the current literature.

In a retrospective study that did account for occult instability, patients with unstable LC1b injuries, compared to patients with stable LC1a injuries, took longer to clear physical therapy, spent more days in the hospital, and were more likely to discharge to a facility [32]. Tucker et al. compared patients with isolated LC1a, LC1b, and intertrochanteric hip fractures and found that patients with LC1b injuries, compared to LC1a injuries, were older (58 vs 39 years), had more comorbidities as demonstrated by an American Society of Anesthesiologist (ASA) classification > 2 (46% vs 7%), had longer hospital stays, and more inpatient opioid use. Comparatively, patients with LC1b injuries and patients with intertrochanteric hip fracture were similar in age, gender, ASA > 2, inpatient opioid use, hospital length of stay, and proportion discharging to facilities, highlighting the similar morbidity between LC1b injuries and hip fractures [16]. Tucker et al. also retrospectively evaluated the early outcomes of patients with LC1b injuries treated with and without operative fixation [58]. The median patient age was 62 years, 77% were female, and a majority (56%) were caused by low-energy mechanisms. Patients with LC1b injuries managed nonoperatively, compared to operatively, were more likely to be in a facility at 2 weeks, more likely to be using assistive devices at 6-week follow-up, and had greater fracture displacement on follow-up radiographs. There were no detectable differences in 2- or 6- week pain scores or opioid use, patient-reported outcome measures, or complications, however. 

High quality prospective evidence from randomized trials and observational cohort studies is lacking to support clinical benefits of early internal fixation over either delayed internal fixation or nonoperative care for older adults with LC1 pelvis fragility fractures. Slobogean prospectively evaluated 95 patients with high-energy minimally displaced LC1 fractures, 65 of whom were randomized to internal fixation or nonoperative care [59]. The mean patient age was 44 years, 63% were women, and the mean ISS score was > 15. Operatively treated patients had a slightly lower pain scores and slightly better Majeed scores over the first 12 weeks, but no difference in length of stay or time to mobilization. The above differences were not considered clinically relevant. This study may have been limited by surgeons’ and patients’ lack of equipoise when an observational cohort was available as an alternative to randomization. The L1FE study was designed to compare non-surgical management with the use of an anterior internal fixator with or without SI screws in patients who were unable to mobilize within 72 h of injury [60]. However, the study was terminated early after failing to achieve sufficient enrollment at the pilot stage primarily because the majority of eligible patients were able to ambulate 6 m at 72 h from injury [15]. The ASSERT (Acute Sacral insufficiency fractuRe augmenTation) Study was a single-site feasibility study comparing surgical and non-surgical management of sacral fractures using sacroplasty with or without SI screw fixation within 7 days of randomization. This study was also terminated early after failing to achieve sufficient enrollment at the feasibility stage based on only 5% of screened patients being eligible to participate and poor adherence to assigned treatment [61]. A feasibility trial comparing health status outcomes excluding patients with fragility fractures resulting from low-energy trauma remains unpublished (ISRCTN10649958) [62].

## Fixation Constructs

Fixation constructs utilized for operative fixation of LC injuries are not standardized and not specific to fragility fractures [39]. In the setting of fragility fractures, the quality of the bone may compromise the stability of fixation. Partially-threaded iliosacral screws often have poor purchase, so fully-threaded transsacral screws are commonly used to by some surgeons to maximize fixation [63, 64]. Presently, there is wide variation in the practice of stabilizing the fracture components of LC pelvic ring disruptions. Some surgeons routinely perform posterior-only fixation with cannulated transsacral or iliosacral screws [54, 59], others perform anterior-only internal fixation [65], and isolated external fixation strategies are reported. [29, 66]

Avilucea et al. described a posterior-first sequence of EUA and internal fixation to determine the need for combined posterior and anterior fixation [67]. LC1 injuries with occult instability on EUA underwent posterior ring fixation with transsacral or iliosacral screws. The EUA was repeated after posterior fixation. If > 1 cm of dynamic displacement persisted, then either antegrade or retrograde ramus screw fixation was performed based on ramus fracture location. Using this protocol 47% of LC1 injuries received posterior-only fixation. All patients with unilateral rami fractures who received posterior-only fixation healed without displacement while all patients with bilateral rami fractures experienced fracture displacement. The authors recommend anterior fixation for patients with bilateral rami fractures.

However, maximizing the stability of fragility fractures with combined anterior and posterior fixation may reduce pain, promote mobilization, and improve the probability of returning home at discharge – established priorities for patients with LC1 pelvis fractures [17]. Anterior fixation constructs include antegrade or retrograde rami screws, external fixation, infix, or open plating [1]. Ellis et al. demonstrated the LC fractures treated with posterior-only fixation had more late fracture displacement than anterior-only or anterior–posterior fixation, which included external fixation (n = 80), rami screws (n = 25), and plating (n = 21) [65]. Tucker et al. evaluated posterior-only versus anterior–posterior fixation of minimally displaced LC1 injuries, a majority of which were fragility fractures [68]. Most of the posterior fixation constructs included a single fully-threaded 7.3 or 8.0 mm cannulated S1 or S2 transsacral screw (21/25). Anterior fixation consisted of 6.5 or 7.3 mm cannulated rami screws, 84% placed retrograde for parasymphyseal fractures. Patients who received posterior-only fixation, compared to anterior–posterior fixation, used more opioids as inpatients, were less likely to clear physical therapy by hospital discharge, and were more likely to discharge to a facility.

The risks of surgical complications must be weighed against the risks of complications secondary reduced mobility in older adults with fragility fractures of the pelvis. Yoon et al. reported 23% of 60 patients with operative LC1 injuries experienced surgical complications and 17% required additional procedures [69]. Complications included loss of reduction ≥ 1 cm (8%), symptomatic hematoma (8%), symptomatic loosening of rami screws (5%), deep infection (1.6%), and an iatrogenic L5 nerve injury (1.6%). All instances of lost fracture reduction occurred in patients with fragility fractures and parasymphyseal rami fractures, highlighting the difficulty of fixation in this setting.

**Fig. 4:**
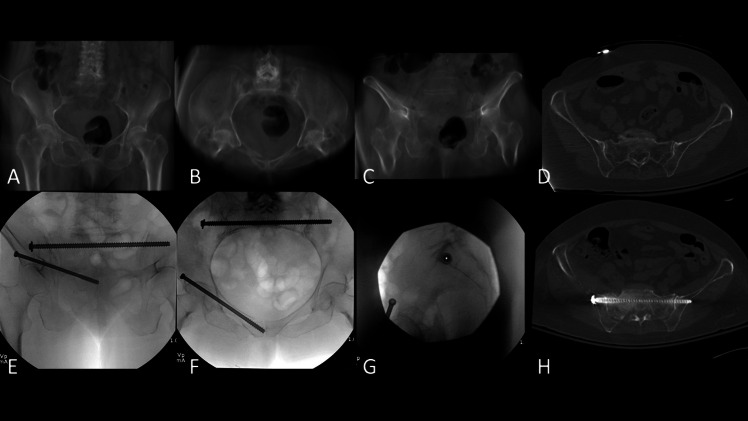
Computed tomography reconstructed **(A)** AP, **(B)** inlet, **(C)** outlet views and **(D)** axial CT image of 71-year-old woman a displaced LC1 fragility fracture of the pelvis. Intraoperative fluoroscopic **(E)** inlet, **(F)** outlet, and **(G)** lateral sacral views and postoperative **(H)** axial CT image demonstrating closed reduction internal fixation with an antegrade ramus screw and a fully-threaded transsacral screw

An important consideration of retrograde rami screw fixation is bicortical fixation, which can be challenging in the setting of narrow bony corridors. Tucker et al. observed that loss of fixation occurred in 18% of retrograde rami screws and was more likely with unicortical fixation [70]. Due to the high rate of retrograde rami screw loosening in comminuted parasymphyseal rami fractures, some surgeons advocate for the use of antegrade screw placement to secure the parasympheal bone with additional screw threads, while others utilize cannulated headless compression screws to gain increased purchase in the small distal rami segment (Fig. 4) [70]. Headless compress screws were biomechanically superior to partially-threaded screws in preventing fracture displacement in a cadaveric study [71].

## Conclusion

LC pelvic ring fragility fractures are common injuries associated with substantial morbidity and mortality rates. Minimally displaced LC injuries are routinely treated nonoperatively, however much of the literature supporting this is based on younger patients with high-energy mechanisms. Early operative treatment of fragility fractures with occult instability on stress imaging may facilitate mobilization and minimize morbidity in this population, but high-quality evidence supporting this practice is lacking. Future research focused on the management of LC pelvic ring fragility fractures is needed and occurring.

Ongoing prospective clinical investigation may clarify this clinical dilemma between operative and nonoperative care. The PIVOT-LC1 (Multicenter Randomized Controlled Trial Comparing Early Internal Fixation Versus NonOperative Care with Early Rehabilitation for LC1 Fragility Fractures of the Pelvis) study will randomize older adults with LC1 fragility fractures of the pelvis to early internal fixation or nonoperative care with early rehabilitation, comparing a composite outcome of mortality, ambulation, and healthy days at home. Two prospective observational cohort studies of internal fixation versus nonoperative care in this population are in development, one including LC1 fractures in older adults and one narrowed to operatively and nonoperatively treated Rommens type II or more severe fragility fractures of the pelvis. A randomized trial of internal fixation versus non-operative treatment comparing pain and ambulation among adults ≥ 60 years of age with LC1 fractures has not yet begun recruiting (NCT05765669). It is expected that results of these studies will further help define orthopaedic management of lateral compression fragility fractures of the pelvis.

## Key References

- Patterson JT, Parry JA, Working ZM, et al. Patient preferences for operative versus nonoperative treatment of LC1 pelvis fracture: a discrete choice experiment. *Journal of Orthopaedic Trauma*. 2022. 10.1097/BOT.0000000000002794.
  - Survivors or LC1 pelvis fracture were more concerned with walking independently, getting out of the hospital within two weeks, and returning to their preinjury living circumstances than with death or reoperation. Patients who received either operative or nonoperative care strongly preferred the treatment their received, indicating satisfaction with their care.
- Parry JA, Funk A, Heare A, et al. An international survey of pelvic trauma surgeons on the management of pelvic ring injuries. *Injury*. 2021;52:2685–2692.
  - Surgeons queried about the management of pelvis fractures found no common ground (moderate to strong agreement in their responses) regarding the diagnosis, characterization, and management of LC1 pelvis fractures.
- Tosounidis T, Kanakaris N, Nikolaou V, et al. Assessment of lateral compression type 1 pelvic ring injuries by intraoperative manipulation: which fracture pattern is unstable? *International Orthopaedics (SICOT)*. 2012;36:2553–2558.
  - The first prospective description of fluoroscopic examination of LC1 pelvis fracture stability as part of an algorithm for treatment by nonoperative management, posterior only fixation, or posterior and anterior internal fixation.
- Rommens PM, Boudissa M, Krämer S, et al. Operative treatment of fragility fractures of the pelvis is connected with lower mortality. A single institution experience. *PLoS One*. 2021;16:e0253408.
  - A previously-described classification system for fragility fractures of the pelvis was validated in clinical practice. Early internal fixation for older adults with these injuries provided a mortality benefit, but was also associated with longer hospital stays are a greater incidence of complications.
- Yoon Y-C, Tucker NJ, Kim YJ, et al. Surgical complications after fixation of minimally displaced lateral compression type 1 pelvic ring injuries. *Eur J Orthop Surg Traumatol*. 2024.
  - Surgical fixation of LC1 fractures is not without risk. The authors identified a 23% incidence of complications, primarily related to loss of reduction, symptomatic hematoma, and loosening of fixation.

## Data Availability

No datasets were generated or analysed during the current study..
